# Principles of Best Practice and Transparency in Scholarly Publishing ver. 4: a Korean translation

**DOI:** 10.12771/emj.2025.00318

**Published:** 2025-03-31

**Authors:** The Committee on Publication Ethics, DOAJ, the Open Access Scholarly Publishing Association, the World Association of Medical Editors

**Affiliations:** The Committee on Publication Ethics, DOAJ, the Open Access Scholarly Publishing Association, the World Association of Medical Editors

## 서론

Committee on Publication Ethics (COPE), Directory of Open Access Journals (DOAJ), the Open Access Scholarly Publishing Association (OASPA)와 World Association of Medical Editors (WAME)는 학술출판에서의 투명성 원칙과 업무 지침을 마련하기 위해 협력한 학술단체이다. 이것은 진행 중인 작업의 네 번째 버전(2022년 9월 15일 출판)으로, 널리 보급되기를 바란다.

‘학술출판에서 투명성 원칙과 업무 지침’은 학술지 특별호(special issue)와 학술대회 자료집을 포함한 모든 출판 자료에 적용해야 한다. 각 학술지의 지침이 표준지침과 다를 경우 편집인은 학술지가 따르는 절차를 명확하게 밝혀야 한다.

또한 이 지침에 따라 출판사와 편집인은 출판의 모든 측면에서 접근성, 다양성, 형평성, 포괄성을 증진할 책임이 있다. 편집인의 결정은 학문의 가치에 바탕을 두어야 한다. 저자의 국적, 민족, 정치적 신념, 인종 또는 종교 등 투고 원고의 가치를 판단하는 데에 영향을 주어서는 안 된다. 학술지는 모든 참여자에게 배타적인 환경을 조성하지 않아야 하며, 참여자 수용 정책을 늘 점검해야 한다.

**Table:** 

학술지 기본 정보	학술지 정책	조직	출판 비용 및 수익원
1. 학술지 표제	7. 출판윤리 정책과 관련 편집 정책	10. 소유권과 운영	13. 게재료
2. 학술지 누리집	8. 전문가심사	11. 편집위원회 또는 자문위원회	14. 기타 수익
3. 발행 간기		12. 편집실/연락처 정보	15. 광고
4. 자료 보존			16. 마케팅
5. 저작권			
6. 라이선스			

## 학술지 기본 정보(Journal Content)

### 1. 학술지명(name of journal)

학술지명은

• 독창적이어야 하고, 다른 학술지와 혼동되지 않아야 한다.

• 저자와 독자들이 학술지의 발행처나 범위를 오인하거나 다른 학술지나 기관과 관련이 있다고 잘못 이해하지 않도록 주의하여야 한다.

### 2. 누리집(website)

• 학술지 누리집은 컴퓨터 바이러스와 악성 프로그램으로부터 사용자를 보호하기 위해 보안을 강화해야 한다.

• 적어도 누리집 URL 프로토콜은 http가 아닌 https(보안 URL protocol)를 사용해야 하며, 모든 트래픽(traffic)은 https를 통해 전송(redirect)되어야 한다. 누리집 관리자는 내용, 구성(presentation)과 응용프로그램(application)에 웹 표준과 표준 윤리지침을 적용해야 한다. 누리집은 독자나 저자에게 오해를 불러일으킬 수 있는 정보를 기술하지 않도록 한다. 누리집에 다른 학술지/출판사의 웹사이트, 디자인, 로고 등을 사용하지 않아야 한다. 다른 누리집의 내용을 복사한 경우, 원본 누리집을 명시해야 한다. 추가로 누리집에는 다음 항목을 분명하게 표시해야 한다.

• 학술지의 목표와 범위

• 목표로 하는 독자층

• 출판할 수 있는 원고의 유형(예시로, 이중게재, 중복게재를 허락하지 않는다는 내용 등 포함)

• 저자 자격 기준(authorship criteria)

• ISSNs (P-ISSN, E-ISSN 모두 기재)

### 3. 발행 간기(publishing schedule)

학술지의 발행 간기를 명확하게 기술하고, 특별한 사정이 없는 한 발행 일정을 지켜야 한다.

### 4. 자료 보존(archiving)

학술지나 발행인이 학술지 발행을 중단하는 경우 학술지 전문 전자 백업과 장기 디지털 보존 계획을 밝혀야 한다. 자료 보존을 위한 기관으로는 PMC와 Keepers Registry에 등록된 기관을 포함된다.

### 5. 저작권(copyright)

• 출판물 저작권 정책은 누리집과 개별 논문에 명시해야 한다.

• 저작권 조건은 누리집 저작권과 별개로 구별되어야 한다.

• 출판된 모든 문헌(HTML과 PDF)의 전문(full text)에 저작권 소유권자를 기재해야 한다.

• 저작권 조건이 별도의 형식으로 설명되어 있는 경우, 누리집에서 누구나 쉽게 찾고 이용할 수 있어야 한다.

### 6. 라이선스(licensing)

• 라이선스 정보를 누리집에 명확히 설명해야 한다.

• 라이선스 조건은 출판된 모든 문헌(HTML, PDF)의 전문(full text)에 표시해야 한다.

• 오픈 액세스로 지정된 모든 콘텐츠는 오픈 라이선스를 사용해야 한다.

• 제3의 저장소에 저자 원고와 출판된 문헌을 게시하는 데 따른 라이선스 정책을 명시해야 한다.

• Creative Commons License (CCL)을 적용하는 경우, 해당 라이선스의 조건을 Creative Commons 웹사이트의 올바른 라이선스 링크로 연결해야 한다.

## 학술지 정책(Journal Practices)

### 7. 출판윤리 정책과 관련 편집 정책(publication ethics and related editorial policies)

학술지는 출판윤리 정책을 누리집에 밝혀야 하며(예: COPE Core Practice 지침), 여기에는 다음 항목을 포함해야 한다.

• 저자와 기여자(contributor) 자격 정책

• 항의와 불만을 처리하는 방법

• 연구윤리 위반 혐의를 처리하는 방법

• 이해관계 정책

• 자료 공유와 재현성 정책

• 연구윤리 준수 정책

• 지적재산권 정책

• 출판 후 논의 정책

• 수정과 취소(철회) 정책

편집인과 발행인은 학술지에 게재된 학술 문헌의 무결성을 보장할 책임이 있다. 표절, 인용 부풀리기, 자료 위조/변조 등 연구윤리 위반 행위가 발생할 때 문제 해결을 위한 정책과 절차를 설명해야 한다. 학술지 정책이나 편집인 성명은 이러한 위반 행위를 장려하거나 의도적으로 허용해서는 안 된다. 편집인이나 발행인이 해당 학술지에서 투고 받은 원고나 이미 발행된 논문에서 연구윤리 위반 행위를 파악한 경우, COPE 가이드라인에 준하는 절차에 따라 처리해야 한다.

### 8. 전문가심사(peer review)

전문가심사란 원고의 주제 분야의 심사자/전문가로부터 조언을 얻는 것이며, 전문가심사자들은 편집팀의 일원이어서는 안 된다. 그러나 전문가심사의 구체적인 방법은 학술지나 분야에 따라 다를 수 있으므로 다음과 같은 내용을 누리집에 기술해야 한다.

• 투고 원고 심사 여부

• 전문가심사 수행 주체(예: 외부 전문가나 편집위원회 구성원)

• 심사 과정의 유형(단일 가림 심사, 양쪽 가림 심사, 공개 심사 등)

• 심사 절차와 관련된 모든 정책은 아래 같은 경우들을 포함할 수 있음

- 저자 추천 심사자를 초빙하는지

- 개인정보가 가려지는지, 가려지는 경우 누가, 누구에게 가려지는지

- 추가 보충자료(supplementary material)가 심사 대상인지

- 심사 내용이 논문과 함께 게시되는지

- 심사자를 명시하는지 여부

• 원고의 최종 결정 과정 및 관련자

• 전문가심사를 받지 않아도 되는 예외적인 특정 논문의 유형

일반적 전문가심사 정책을 따르지 않는 경우라면 해당 논문이 어떤 심사를 받았는지 밝혀야 한다. 학술지는 처음 투고할 때 해당 원고의 수락을 보장해서는 안 된다. 수락된 원고는 심사 기간에 대해 명시된 대로 출판되어야 한다. 심사가 지연될 때는 저자에게 그 이유를 알려야 하며, 저자가 원한다면 원고를 철회(withdrawal)할 기회를 주어야 한다. 출판 일자는 모든 출판 논문에 공표해야 하고, 접수 일자와 채택 일자를 함께 기재하는 것이 바람직하다.

### 9. 접근성(access)

회원가입, 구독이나 유료 논문과 같이 모든 사람이 자유롭게 접근할 수 없는 온라인 콘텐츠가 있는 경우 접근 방법을 명확하게 설명해야 한다. 인쇄본을 구독할 수 있는 경우라면 구독료를 명시해야 한다.

## 조직(Organization)

### 10. 소유권과 운영(ownership and management)

• 학술지 소유권과 운영 관리 정보는 누리집에 밝혀야 한다.

• 투고자나 편집인이 학술지 소유자의 특성에 대해 오해할 수 있는 기관명은 사용하지 않도록 한다.

• 학술지가 학회, 기관이나 스폰서에 소속되어 있는 경우 가급적 누리집 링크(학회, 기관 나 스폰서)를 제공해야 한다.

### 11. 자문기관(advisory body)

학술지에는 Aims and Scope에 명시된 주제 분야 전문가로 구성된 편집위원회나 자문위원회가 있어야 한다.

• 위원의 이름과 소속을 학술지 누리집에 기재한다.

• 위원회 명단은 최신 정보여야 하며, 위원은 위원회 활동에 동의해야 한다.

• 위원의 최신 정보를 주기적으로 확인하여 갱신해야 한다.

### 12. 편집실/연락처 정보(editorial team/contact information)

학술지는 누리집에 메일을 포함한 편집사무실 연락정보, 편집위원들의 이름과 소속을 반드시 제시해야 한다.

## 출판 비용 및 수익원(Business Practices)

### 13. 저자 비용 또는 게재료(author fees)

• 게재료(논문 처리 비용, 페이지당 비용, 편집 비용, 언어 교정 비용, 컬러 인쇄 비용, 투고 비용, 회비, 기타 부가 비용 등)가 부과된다면 누리집에 그 비용을 명확히 표시한다.

• 게재료가 없다면 이를 분명하게 밝힌다.

• 게재료에 대한 정보는 쉽게 찾을 수 있어야 하며, 투고 과정 중 앞 부분에서 제공해야 한다.

• 향후 게재료를 부과할 가능성이 있는 경우 이를 명시한다.

• 게재료 면제 제도가 있으면 면제 대상이나 자격, 신청 시기, 방법 등의 정보를 밝힌다.

• 게재료나 면제 여부가 편집위원회의 심사와 게재 판정에 영향을 미치지 않아야 하며, 이를 명기한다.

### 14. 기타 수익(other revenue)

사업 모델 또는 수익원을 누리집에 명시해야 한다. 사례로는 저자 비용(13번 참조), 구독, 후원금과 보조금, 광고(15번 참조), 별쇄본, 부록, 특별호 등을 포함한다. 사업 모델 또는 수익원(예: 별쇄본 수익, 부록, 특별 호, 후원)이 편집위원회의 심사와 게재 판정에 영향을 미치지 않아야 한다.

### 15. 광고(advertising)

학술지는 광고 게재 여부를 명시해야 한다. 광고를 고려한다면 아래와 같은 광고 정책을 밝혀야 한다.

• 어떤 형태의 광고를 고려할지

• 누가 광고를 수락할지

• 논문 내용이나 독자의 이용 형태에 따라 광고를 연동할지 아니면 무작위로 노출할지 광고가 편집위원회의 의사 결정과 관련되어서는 안 되며, 논문 내용과 무관해야 한다.

### 16. 마케팅(direct marketing)

원고 의뢰를 포함하여 학술지를 대신해 수행하는 모든 직접 마케팅 활동은 적절하고, 대상이 명확해야 하며, 지나치지 않아야 한다. 발행인이나 학술지 정보를 사실대로 제공하여 독자 또는 저자에게 오해를 불러일으키지 않아야 한다.

## Version History

• This is Version 4.0 of the Principles of Transparency and Best Practice in Scholarly Publishing

• Version 3.0: January 2018

• Version 2.0: June 2015 (on the OASPA website)

• Version 1.0: December 2013 (on the OASPA website)

## 영국출판윤리위원회(COPE, https://publicationethics.org)

COPE는 출판윤리의 모든 측면, 특히 연구 및 출판윤리 위반 사례를 처리하는 절차를 편집인과 발행인에게 제공한다. 또한 회원들이 개별 사례를 토론할 수 있는 장을 제공한다. COPE가 개별 사례를 조사하지는 않지만 적절한 권위자(일반적으로 연구기관 또는 고용주)가 해당 사례를 조사할 수 있도록 편집인에게 권고한다. 모든 COPE 회원은 처리 기준에 명시된 출판윤리에 관한 COPE 원칙을 적용해야 한다.

## 오픈 액세스 저널 디렉토리(DOAJ, https://doaj.org)

DOAJ는 (1) 오픈 액세스 학술지에 대한 신뢰할 수 있는 누리집 정보를 관리, 유지 및 개발하고, (2) 회원 목록 내 각 항목이 표준을 준수하는지 확인하며, (3) 오픈 액세스 학술지의 가시성, 유통, 검색 및 선호도를 증가시키고, (4) 연구자, 도서관, 대학, 연구비 제공 기관, 기타 이해당사자가 DOAJ에서 제공하는 정보와 서비스의 혜택을 누릴 수 있도록 하며, (5) 오픈 액세스 학술지가 도서관 및 서지정보 제공자(aggregator) 서비스에 통합되는 것을 편리하게 하며, (6) 발행인과 학술지가 전자출판 표준을 준수할 수 있도록 지원하고, 나아가 (7) 학술 교류와 출판 시스템이 과학, 고등교육, 산업, 혁신, 사회와 인류에 봉사하는 모델이 될 수 있도록 지원한다. 위와 같은 활동을 통해 DOAJ는 동일한 목표를 향해 노력하는 모든 관련 당사자와 협력할 것이다.

## 오픈 액세스 학술출판 협회(OASPA, https://oaspa.org)

OASPA는 분야를 막론한 전 세계 오픈 액세스 발행인의 이익을 대변하기 위해 2008년에 설립된 동업자 단체이다. OASPA의 목표는 오픈 액세스 출판을 지원하는 비즈니스 모델, 도구와 표준을 개발함으로써 회원과 학술 커뮤니티의 이익을 위해 지속 가능하고 발전된 미래를 보장하는 것이다. 이런 사명으로 정보를 교환하고 표준을 수립하며, 더 나아가 사업 모델, 홍보, 교육, 혁신을 촉진하고 있다.

## 세계의학편집인협의회(WAME, http://www.wame.org)

WAME는 편집인 간의 협력과 소통을 증진하고, 편집 수준을 향상시키고, 교육, 자기 성찰, 자기 관리를 통하여 의학학술지 편집의 전문성을 증진하며, 의학 편집의 원칙 및 실무에 관련한 연구를 장려하고자 의학 학술지 편집인들이 자발적으로 만든 국제 비영리 단체이다. WAME는 의학학술지 편집인의 업무 처리에 유용한 정책과 권고안을 마련하고, 회원 편집인을 위한 교재를 개발하고 있다.

